# Palladium-based pseudohomogeneous catalyst for highly selective aerobic oxidation of benzylic alcohols to aldehydes

**DOI:** 10.1038/s41598-023-49526-y

**Published:** 2024-01-04

**Authors:** Homa Targhan, Aram Rezaei, Alireza Aliabadi, Ali Ramazani, Zhefei Zhao, Huajun Zheng

**Affiliations:** 1 Nano Drug Delivery Research Center, Health Technology Institute, Kermanshah University of Medical Sciences, Kermanshah, Iran; 2 Pharmaceutical Sciences Research Center, School of Pharmacy, Health Institute, Kermanshah University of Medical Sciences, Kermanshah, Iran; 3 Department of Chemistry, University of Zanjan, Zanjan, 45371-38791 Iran; 4 Department of Applied Chemistry, Zhejiang University of Technology, Hangzhou, 310032 China

**Keywords:** Catalysis, Green chemistry, Materials chemistry, Organic chemistry

## Abstract

This study presents a novel class of pseudohomogeneous catalysts (PHC) based on carbon quantum dots functionalized with terpyridine ligands (CQDs-Tpy) to immobilize and stabilize palladium nanoparticles (Pd NPs). Extensive characterization techniques clearly confirmed the successful stabilization of Pd NPs on CQDs-Tpy. The effectiveness of the catalyst was demonstrated in the selective aerobic oxidation of primary and secondary of benzylic alcohols to aldehydes in the absence of additives and phase transfer catalyst (PTC). Remarkably, the reactions predominantly yielded aldehydes without further oxidation to carboxylic acids. By employing low catalyst loadings (0.13 mol%), high conversions (up to 89%) and excellent selectivity (> 99%) of the aldehyde derivatives were achieved. Moreover, the CQDs-Tpy/Pd NPs catalyst displayed suitable catalytic activity and recyclability, offering potential economic advantages. This promising approach opens up new opportunities in the field of catalysis for designing subnanometric metal-based PHCs.

## Introduction

The oxidation of alcohols is the efficient and significant reaction pathway for the achievement of the carbonyl-containing compounds. The carbonyl-containing compounds have always been dominant compounds because they have frequently been utilized as precursors in the synthesis of a plethora of high-value product^1–5^. Despite the large number of undesirable environmental and economic effects continue to use stoichiometric oxidations such as Dess-Martin periodinane (DMP), Swern, Pfitzner–Moffatt, Corey-Kim, Jones, Collins, Oppenauer and pyridinium chlorochromate (PCC) reagents^1,6^. Recent ideas on catalytic research led to the usage of environmentally benign oxidant such as molecular oxygen and hydrogen peroxide (H_2_O_2_) in combination with active and selective recyclable catalysts based on transition-metal (such as Pd, Ru, Ir, Rh, Fe, Cu, Pt, Au, Ag, etc.,). These developments have paved the way for more sustainable oxidation reactions, addressing the drawbacks associated with traditional stoichiometric oxidations^7–11^ In recent years, substantial advancements have been achieved in the realm of developing cost-effective heterogeneous catalysts for selective oxidation, with a dedicated focus on the principles of green chemistry. Nevertheless, this field remains a captivating and extensively explored subject within the scientific literature, attracting significant attention and interest^12–14^. Despite the introduction of catalytic systems with high activity and selectivity for this type of transformation, their industrial applications are hindered by issues related to recovery, recyclability, and environmental concerns. Furthermore, the complexity and cost of catalyst manufacturing, as well as the use of toxic and hazardous substances, restrict their widespread utilization in large-scale quantities. In light of these challenges, heterogeneous Pd catalysis emerges as a promising avenue of research to overcome these obstacles^15^.

In the past two decades, nanocatalysts have indeed played a pivotal role in advancing synthetic organic transformations. Heterostructure nanocatalysts are typically prepared by dispersing and stabilizing nano-scale active materials on a solid support. Extensive evidence has demonstrated that the shape, size, composition, and structure of these nanocatalysts profoundly influence their catalytic activity, selectivity, and stability^16^. Pd based heterogeneous catalysts have demonstrated remarkable catalytic activity, selectivity, and stability, surpassing conventional catalysts. As a result, they are emerging as highly intriguing catalysts for selective oxidation reactions of alcohols. The ongoing research investigating the utilization of Pd-based heterogeneous catalysts in this context highlights their crucial significance in this field^17,18^. A wide range of heterogeneous supports, including polymers^19,20^, carbon nanotubes^21^, graphene oxide sheets^22^, Fe_3_O_4_ nanoparticle^23^, metal–organic framework (MOF)^24,25^, alumina^26^, silica^27^, and more, have been successfully employed for the stabilization of Pd NPs. Among the various techniques used for stabilizing Pd NPs, the utilization of ligands has gained significant popularity^28^ Terpyridine-based ligands, which belong to the class of tridentate nitrogen ligands, have been extensively studied for their ability to effectively stabilize noble metal nanoparticles. The strong interaction between the metal and the nitrogen atom is believed to be the key factor contributing to the effectiveness of terpyridine-based ligands as stabilizing agents for metal nanoparticles^5,29,30^ Furthermore the terpyridine-based ligands possess a significant π-conjugation, which contributes to their favorable electrical conductivity properties^31^.

Carbon quantum dots (CQDs), an emerging allotrope of the carbon family, have exhibited tremendous potential as catalysts in chemical transformations due to their promising physicochemical properties^32,33^. Interestingly, CQDs can serve as a bridge between the realms of heterogeneous and homogeneous catalysis, owing to their subnanometric dimensions. Therefore, they can be regarded as pseudohomogeneous catalysts (PHCs)^34–37^. On the other hand, the utilization of CQDs as a support for metal immobilization presents a remarkable prospect, as the catalytically active entities can remain suspended indefinitely owing to the minute particle size of CQDs and the deliberate inclusion of tailored functional groups. Moreover, the thin layer of CQDs encompasses a diverse array of reactive oxygen functional groups on its surface, thereby imparting substantial aqueous solubility and considerable potential for facile modifications^38^. In essence, techniques for surface modification hold great promise in terms of altering the surface properties of CQDs to suit specific applications, thereby opening up exciting avenues for exploration^35,36,39^.

As part of our ongoing investigations aimed at developing environmentally-friendly catalysts for organic transformations, the initial phase of our study involved the synthesis of CQDs functionalized with terpyridine ligands (CQDs-Tpy) using a straightforward one-step method. Subsequently, Pd NPs were subsequently prepared by reducing the Pd^2^^+^ in the presence of CQDs-Tpy in ethanol as a green reducing agent. In this process, the CQDs-Tpy not only facilitated the reduction process but also acted as stabilizing agents for the resulting Pd NPs. The resulting CQD-Tpy/Pd NPs composite was utilized as a homo/heterogeneous catalyst for the highly-selective aerobic oxidation of alcohols without using any additive or PTC, leading to the formation of their corresponding aldehydes.

## Experimental section

### Synthesis of CQDs-Tpy/Pd NPs

In a typical experiment, 4-([2,2′:6′,2″-terpyridin]-4′-yl)benzoic acid (0.5 mmol) and citric acid (0.5 mmol) and were dissolved into 20 mL DMF. Then 1 mL NH_3_ was added and the resulting solution was transferred into a Teflon-lined stainless-steel autoclave. After heating at 200 °C for 5 h, the autoclave then cooled to room temperature naturally. The obtained solution was filtered through a dialysis membrane (100 Da) for 72 h to remove the remaining molecular precursors and then lyophilized to obtained the CQDs-Tpy (150 mg).

A solution of 100 mg of CQDs-Tpy was thoroughly dissolved in 5 mL of ethanol. Subsequently, PdCl_2_ weighing 5 mg (0.028 mmol) was introduced into the resulting solution, which was then subjected to reflux conditions for a duration of 24 h. During this period, the initially bright brown solution underwent a notable colour change, transitioning to a dark gray hue, indicating the successful formation of Pd NPs. The resulting mixture was subjected to centrifugation at a speed of 14,000 rpm, followed by a vacuum drying process lasting 24 h, obtaining ~ 95 mg CQDs-Tpy/Pd NPs powder. Quantitative analysis using inductively coupled plasma (ICP) revealed that 0.0218 mmol of Pd was effectively loaded onto the initial 100 mg of CQDs-Tpy, corresponding to a weight percentage of 2.31%.

### General procedure for aerobic oxidation of alcohol derivatives

CQDs-Tpy/Pd NPs (1.2 mg/mL, 0.13 mol%) was added to a mixture of alcohol (1.0 mmol) and K_2_CO_3_ (1.0 mmol) in EtOH:H_2_O mixture (1:1 v/v, 5 mL) at 90 °C. The reaction mixture was then stirred. The progress of the reaction was monitored by TLC. After the completion of the reaction, the contents were centrifuged to separate the catalyst from the production mixture and the solvent was evaporated (Table 2).

## Results and discussion

### Synthesis and characterization of the catalyst

**Figure 1:**
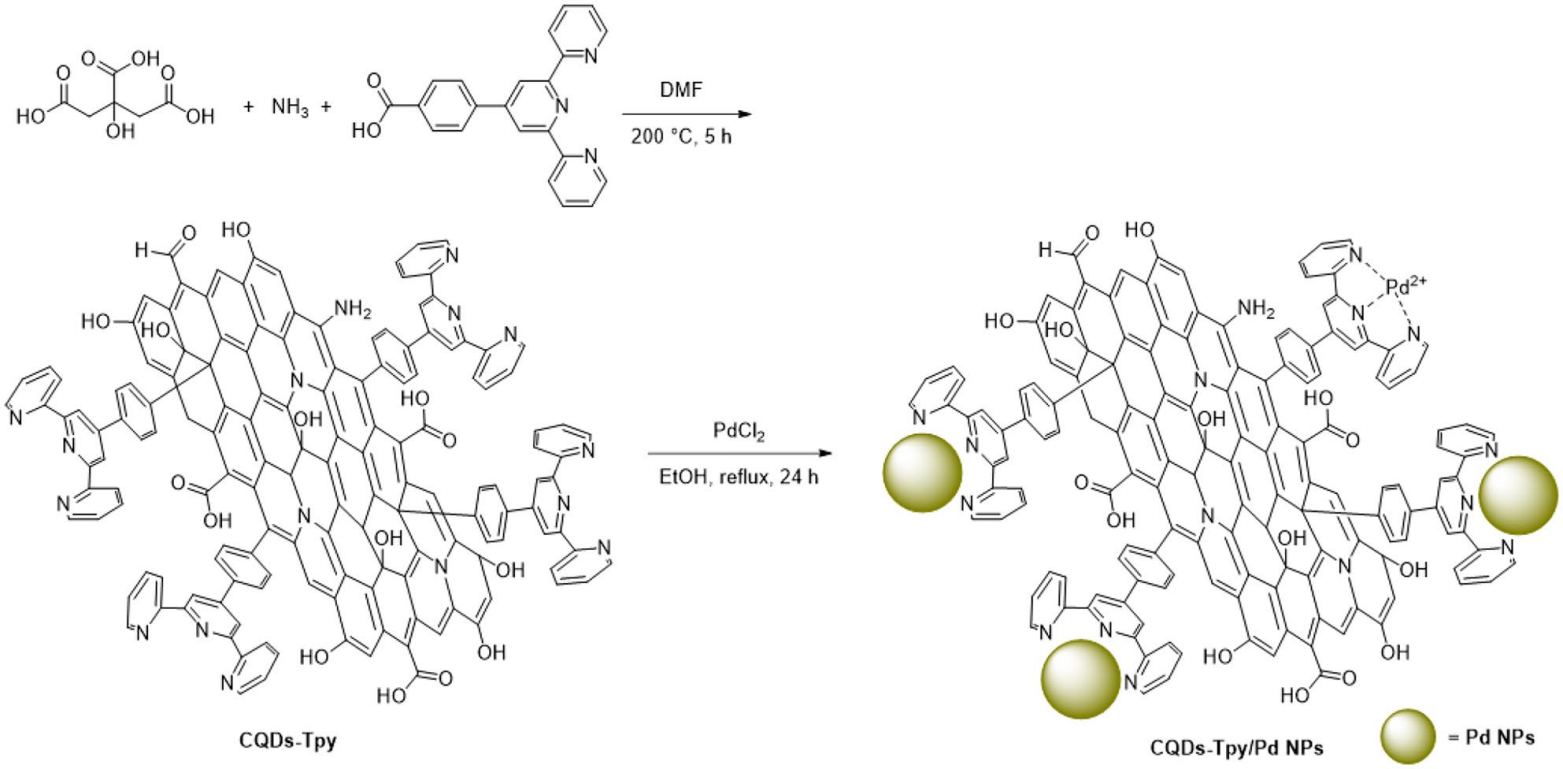
Schematic representation of synthesis procedure of CQDs-Tpy/Pd NPs.

The synthesis of Pd NPs involve reducing Pd(II) ions using a reducing agent in the presence of a dispersing/capping agent. This process allows for the formation of Pd(0) nanoparticles with precise dimensions and defined shapes. Commonly used chemical methods employ excessive amounts of a reducing agent such as sodium borohydride, hydrazine, formaldehyde, or hydrogen^40^. It has been observed that tridentate nitrogen ligands, including ligands based on terpyridine, enhance the dispersion and stability of the metal nanoparticles due to the strong interaction between metal and nitrogen^19^. Alternatively, CQDs have been widely studied as reducing agents for metal salts^41^. In this study, CQDs containing a terpyridine ligand were employed as both reducing and stabilizing agents. The presence of the terpyridine ligand on the surface of the CQDs-Tpy facilitated the binding of Pd(II) ions to the CQDs, enabling their reduction to Pd(0). Consequently, the CQDs-Tpy served effectively as a support for immobilizing Pd nanoparticles without the need for an additional reducing agent. The synthesis of CQDs-Tpy/Pd nanoparticles involved the reduction of Pd(II) ions using as prepared CQDs-Tpy in ethanol, as illustrated in Fig. 1.

The synthesized CQDs-Tpy and CQDs-Tpy/Pd NPs were subjected to characterization using various techniques, including Fourier transform infrared spectroscopy (FTIR), energy-dispersive X-ray spectroscopy (EDX), X-ray diffraction (XRD), X-ray photoelectron spectroscopy (XPS), transmission electron microscopy (TEM), fluorescence spectroscopy, and UV–vis reflectance spectroscopy. The results of the characterization analysis are presented below.

**Figure 2:**
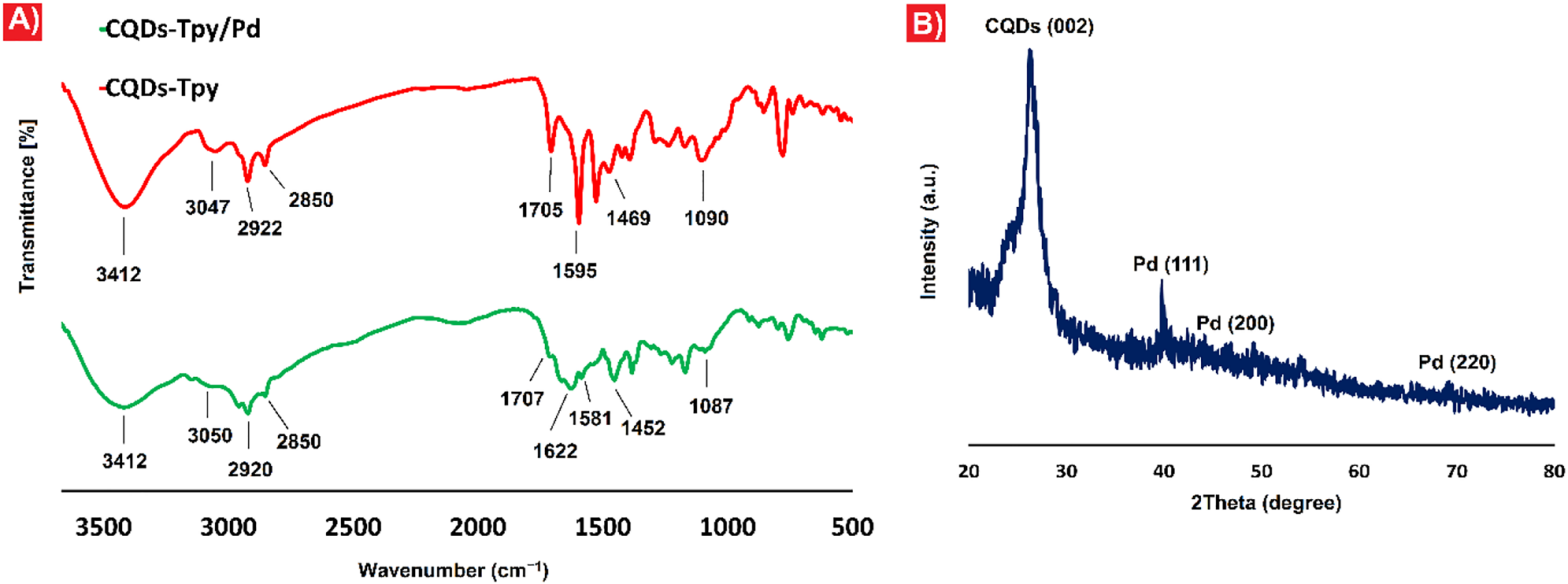
**A** FT-IR spectra of CQDs-Tpy (red) and CQDs-Tpy/Pd NPs (green), (**B**) XRD pattern of CQDs-Tpy/Pd NPs.

Figure 2A shows FT-IR spectra of the prepared CQDs-Tpy and CQDs-Tpy/Pd NPs. The broad absorption peak of OH at 3412 cm^−1^, the stretching vibration band of C=O in carboxylic acid functional group at 1705 cm^−1^, the stretching vibration bands of C=N in pyridine rings at 1595 cm^−1^ and the stretching vibrations C–O at 1090 cm^−1^ confirm the existence of -OH, − COOH and terpyridine ligands at the surface of the CQDs (Fig. 2A). In the case of CQDs-Tpy/Pd NPs, the broad absorption peak at 3412 cm^−1^ corresponds to -OH bands. Two absorption peaks at 2920 and 2850 cm^−1^ are attributed to the sp^3^ C–H stretching vibrations and the band observed at 3050 cm^−1^ is associated with C–H aromatic stretching vibration. The signal at 1707 cm^-1^ can be correspond to the stretching vibration of C=O bonds. The presence of C=N in pyridine rings is confirmed by the peak at 1581 cm^-1^. The C=N bands of pyridine rings in CQDs-Tpy/Pd NPs is shifted to a lower frequency (1581 cm^−1^) in the FT‐IR spectrum compared to that of CQDs-Tpy (1595 cm^−1^). The lowering in frequency of the C=N peak can represent the interaction of pyridine rings with Pd (Fig. 2A)^36^.

Figure 2B illustrates the XRD pattern of CQDs-Tpy/Pd NPs. The XRD pattern exhibits a prominent peak at approximately 2θ = 26°, corresponding to the (002) plane of amorphous CQDs^42^. Additionally, several peaks are observed at 2θ values of 39.77°, 46.67°, and 69.07°, which can be indexed to the (111), (200), and (220) planes, respectively, aligning with the structure of Pd NPs^43^.

**Figure 3:**
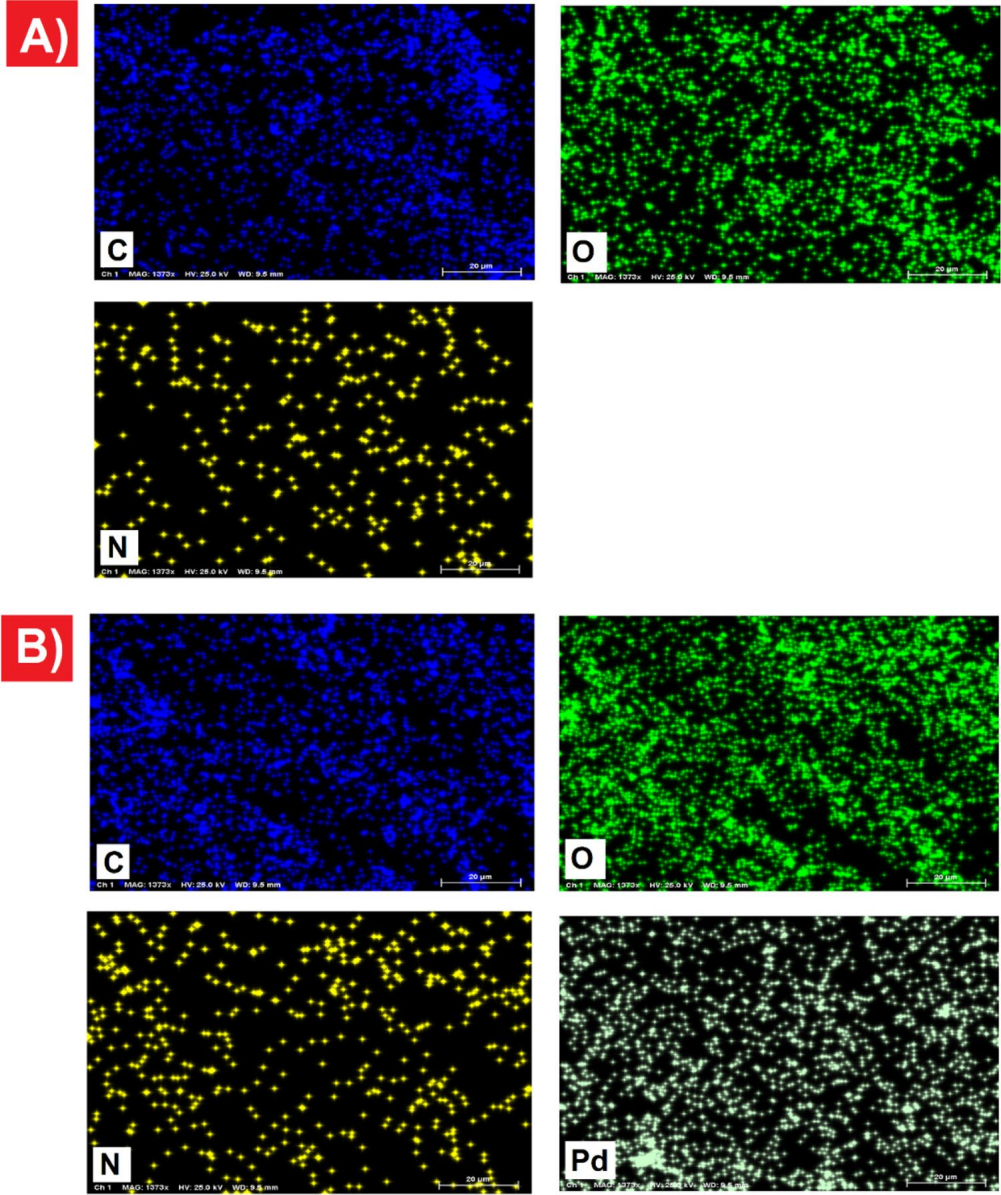
(**A**) EDX mapping of CQDs-Tpy, (**B**) CQDs-Tpy/Pd NPs.

In order to investigate the distribution and chemical composition of CQDs-Tpy and CQDs-Tpy/Pd NPs, EDX mapping analysis was conducted. The mapping images indicate the presence of carbon, oxygen, and nitrogen elements throughout the area of CQDs-Tpy (Fig. 3A). In the case of CQDs-Tpy/Pd NPs, the EDX mapping clearly demonstrates the presence of Pd in addition to carbon, oxygen, and nitrogen. Furthermore, the EDX elemental map images exhibit the uniform distribution of all elements in the prepared CQDs-Tpy/Pd NPs sample (Fig. 3B)^3,36,37^.

**Figure 4:**
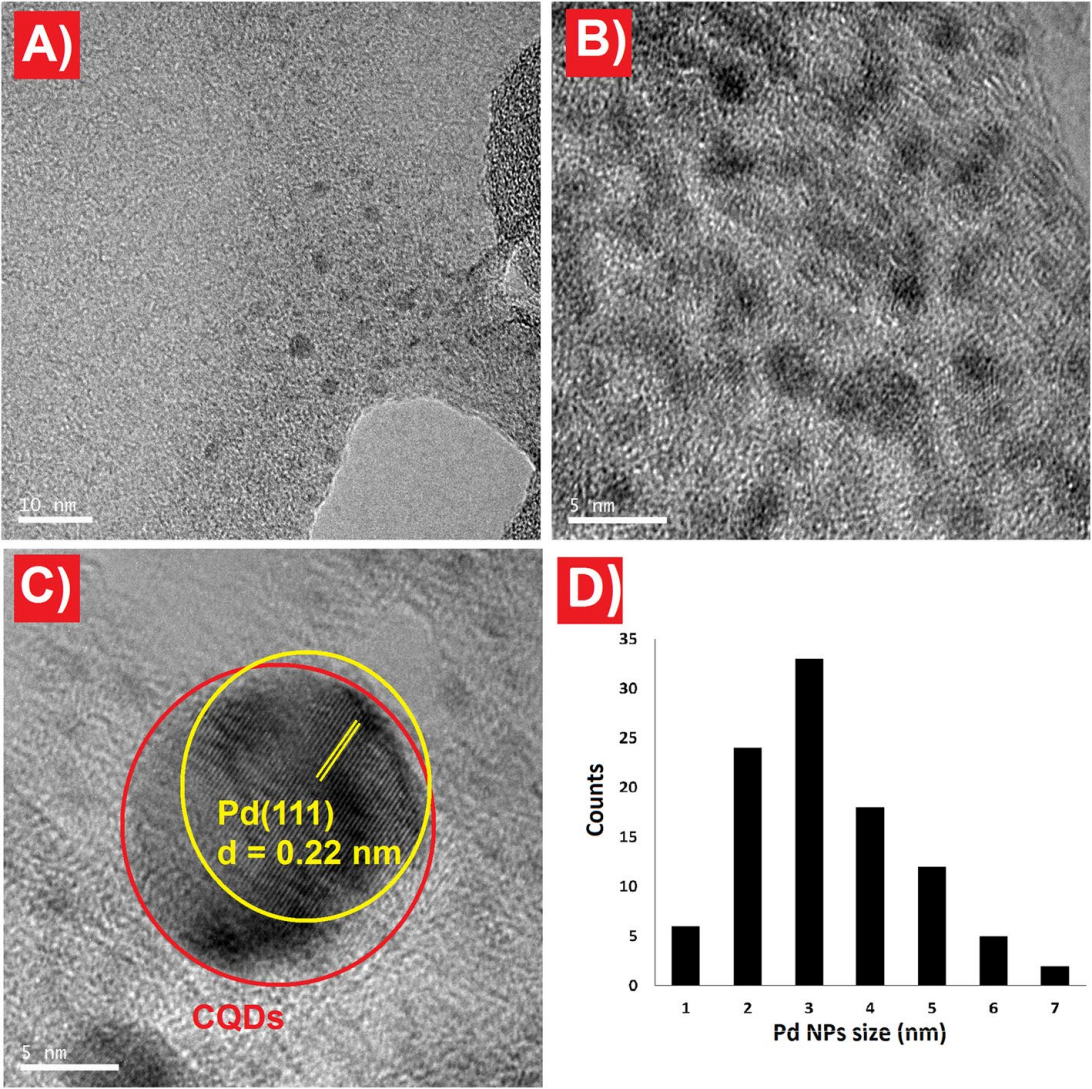
TEM image of CQDs-Tpy sample (**A**), HRTEM images of CQDs-Tpy/Pd NPs (**B** and **C**), particle size distribution histograms of a Pd NPs (**D**).

TEM analysis was employed to investigate the morphological characteristics, including shape, size, and particle distribution of the CQDs-Tpy sample. The TEM image clearly reveals the uniform distribution of spherical CQDs-Tpy particles, primarily measuring less than 5 nm in diameter (Fig. 4A). Subsequently, the synthesized CQDs-Tpy/Pd NPs sample was examined using HRTEM to confirm the presence of Pd nanoparticles and determine their size and shape (Fig. 4B and C). The HRTEM analysis confirms that the as-synthesized CQDs-Tpy/Pd NPs exhibit a spherical-like shape with a diameter of less than 10 nm. Furthermore, the presence of Pd is authenticated by observing its lattice spacing, where a spacing of 0.22 nm corresponds to the (111) plane of Pd (Fig. 4C)^44^. Figure 4D shows the histogram of Pd NPs size distribution. The Pd nanoparticles have a mean diameter of less than 5 nm, which indicates the high monodispersion of Pd NPs on the surface of CQDs-Tpy.

**Figure 5:**
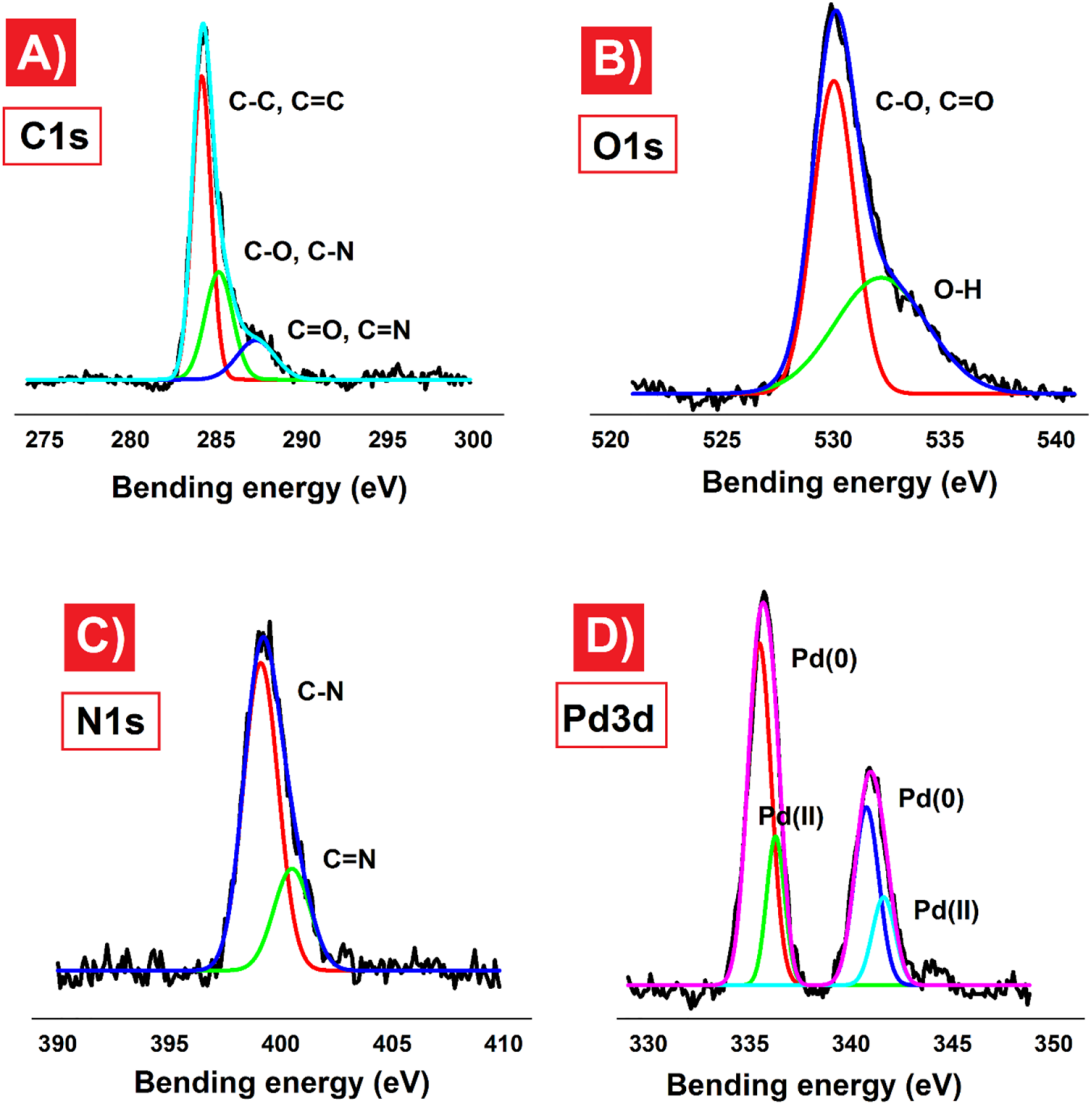
High resolution XPS spectra of CQDs-Tpy/Pd NPs sample (**A**) C1s, (**B**) O1s, (**C**) N1s, and (**D**) Pd3d.

XPS analysis was utilized to explore the atomic chemical states of the synthesized CQDs-Tpy/Pd NPs sample (Fig. 5). The full XPS spectra of CQDs-Tpy/Pd NPs clearly indicate the presence of carbon, oxygen, nitrogen, and palladium (Figure S1). The C1s spectrum exhibits peaks at binding energies (BEs) of 284.25 eV (corresponding to C–C and C=C), 285.36 eV (corresponding to C-O and C-N), and 287.40 eV (corresponding to C=N and C=O) (Fig. 5A). Analysis of the O1s spectrum reveals peaks at BEs of 530.01 eV (assigned to C-O and C=O) and 532.05 eV (assigned to O–H) (Fig. 5B). The N1s spectrum displays peaks at 399.40 eV and 400.62 eV, corresponding to C-N and C=N, respectively (Fig. 5C)^35^. The XPS results confirm the presence of both Pd (0) and Pd (II) species on the CQDs-Tpy/Pd NPs (Fig. 5D). The peaks observed at 335.52 eV (Pd 3d5/2) and 340.75 eV (Pd 3d3/2) are assigned to Pd (0), while the weaker peaks at 336.35 eV (Pd 3d5/2) and 341.78 eV (Pd 3d3/2) are attributed to Pd (II) species present on the CQDs-Tpy/Pd NPs^45^. Based on the XPS results, Pd nanoparticles dominate the Tpy/Pd NPs sample, with only a small amount of Pd (II) ions detected. These Pd (II) ions are likely complexed with terpyridine ligands through the formation of two five-membered rings (Fig. 1).

**Figure 6:**
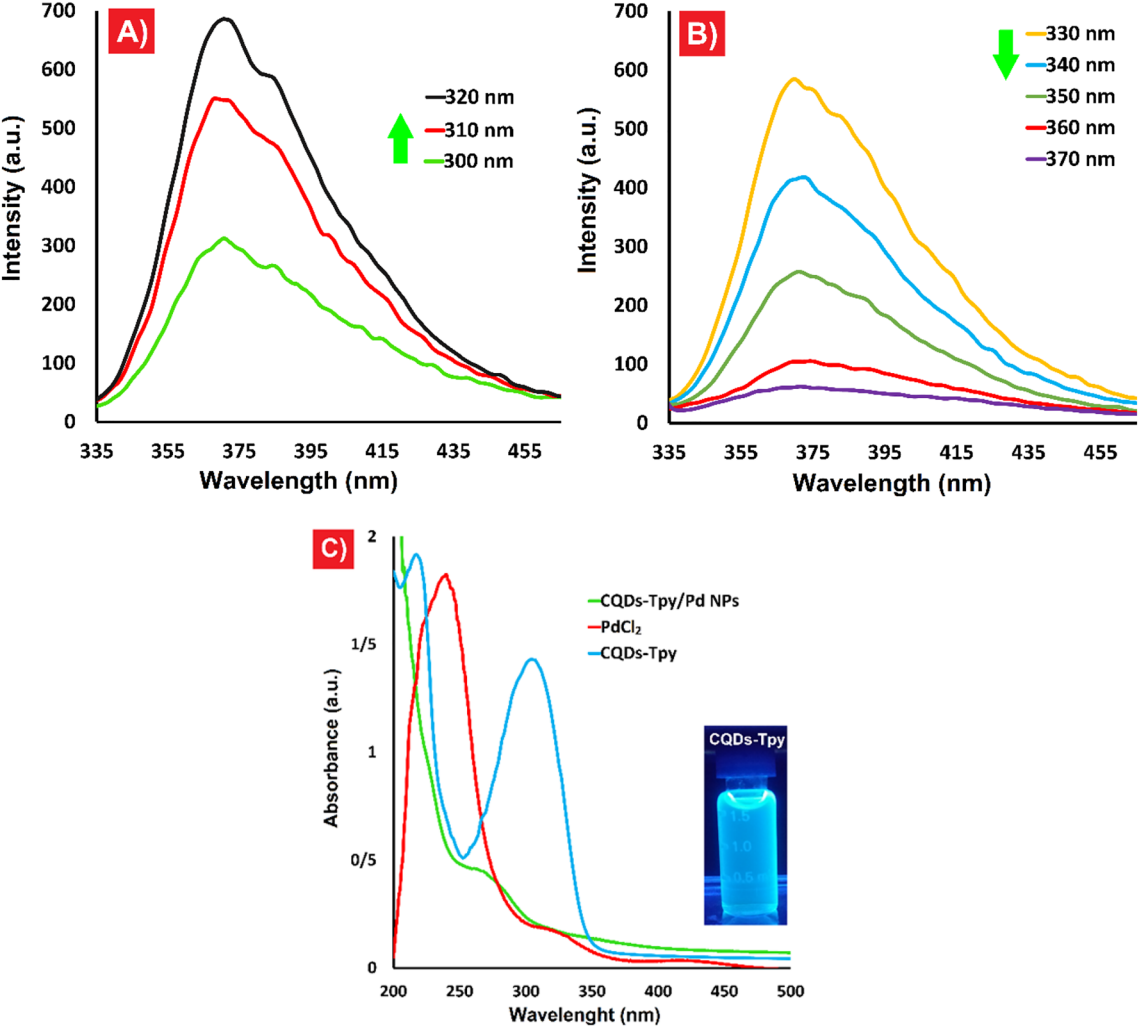
(**A** and **B**) Fluorescence spectrum of CQDs-Tpy at various excitation wavelengths (300–370 nm); (**C**) UV spectra of CQDs-Tpy, PdCl_2_, and the CQDs-Tpy/Pd NPs, the inset showed the emission of CQDs-Tpy dissolved in deionized water under 365 nm UV irradiation.

The fluorescence response of the CQDs-Tpy was examined at various excitation wavelengths ranging from 300 to 370 nm. The fluorescence intensity of the CQDs-Tpy sample exhibited an initial increase (Fig. 6A) followed by a decrease (Fig. 6B), consistent with the fluorescence behavior typically observed in CQDs. Figure 6A illustrates a strong fluorescence peak centered at 372 nm, with maximum emission, when excited at 320 nm. Additionally, the UV–vis study confirmed the formation of CQDs-Tpy/Pd NPs as evidenced by the absence of absorbance from its precursors (Fig. 6C). The characteristic absorbance peaks of CQDs at 225 nm and 300 nm, along with a distinct peak at 241 nm and a broad peak between 400 and 450 nm, indicative of the presence of Pd (II) ions, were obscured in the UV–Vis absorption spectra of CQDs-Tpy/Pd NPs^41^. These findings strongly support the formation of Pd nanoparticles on the surface of CQDs. Furthermore, a photograph of an aqueous dispersion of CQDs-Tpy under 365 nm UV irradiation is presented in Fig. 6C.

### Applications of CQDs-Tpy/Pd NPs for aerobic oxidation of alcohols

**Figure 7:**
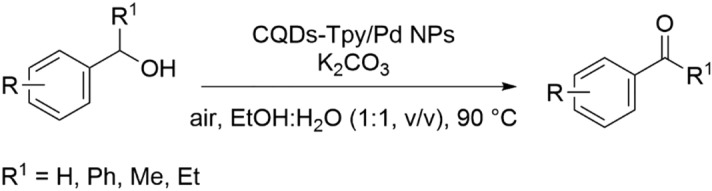
Selective aerobic oxidation of primary and secondary alcohols.

The oxidation of alcohols is considered one of the most extensively employed methods for the preparation of carbonyl compounds, including aldehydes, ketones, and carboxylic acids. However, achieving high selectivity in alcohol oxidation reactions to yield a single desired product remains a significant challenge^2,46^. In this study, we propose the utilization of CQDs-Tpy/Pd NPs as a reusable and efficient PHC for the selective aerobic oxidation of primary and secondary alcohols to aldehydes. The reactions were carried out in an ethanol–water mixture (1:1 v/v) at room temperature, providing a practical and environmentally friendly approach (Fig. 7) without using any additive or PTC.

The catalytic performance of CQDs-Tpy/Pd NPs in the aerobic oxidation of alcohols to aldehydes was evaluated using air as the oxidant. Initially, benzyl alcohol was selected as a standard substrate to assess the effectiveness of the prepared CQDs-Tpy/Pd NPs (Table 1). Various experimental parameters, such as catalyst amount, oxidant, solvent, temperature, and K_2_CO_3_/benzyl alcohol molar ratio, were systematically investigated to determine the optimal conditions. The impact of these reaction parameters on the oxidation of benzyl alcohol (1 mmol) with CQDs-Tpy/Pd NPs is summarized in Table 1.

**Table 1:** Optimization of reaction conditions for the synthesis of benzaldehyde.


Entry^a^	Catalyst (mg/mL)	Oxidant	K_2_CO_3_ (mmol)	Solvent	T (°C)	Yield (%)
1	–	Air	1	EtOH/H_2_O	90	–
2	CQDs-TPy (1.2)	Air	1	EtOH/H_2_O	90	–
3	CQDs-TPy/Pd NPs (0.4)	Air	1	EtOH/H_2_O	90	53
4	CQDs-TPy/Pd NPs (0.8)	Air	1	EtOH/H_2_O	90	69
5	CQDs-TPy/Pd NPs (1.2)	Air	1	EtOH/H_2_O	90	85
6	CQDs-TPy/Pd NPs (1.2)	Air	1	EtOH/H_2_O	r.t	52
7	CQDs-TPy/Pd NPs (1.2)	Air	1	EtOH/H_2_O	50	66
8	CQDs-TPy/Pd NPs (1.2)	Air	1	Toluene	90	12
9	CQDs-TPy/Pd NPs (1.2)	Air	1	H_2_O	90	37
10	CQDs-TPy/Pd NPs (1.2)	Air	1	CH_3_CN/ H_2_O	90	74
11	CQDs-TPy/Pd NPs (1.2)	Air	–	EtOH/H_2_O	90	37
12	CQDs-TPy/Pd NPs (1.2)	Air	2	EtOH/H_2_O	90	72
13	CQDs-TPy/Pd NPs (1.2)	H_2_O_2_ (1 eq.)	1	EtOH/H_2_O	r.t	65
14	CQDs-TPy/Pd NPs (1.2)	H_2_O_2_ (2 eq.)	1	EtOH/H_2_O	r.t	75
15	CQDs-TPy/Pd NPs (1.2)	H_2_O_2_ (3 eq.)	1	EtOH/H_2_O	r.t	82
16	CQDs-TPy/Pd NPs (1.2)	H_2_O_2_ (3 eq.)	1	EtOH/H_2_O	50	91

^a^Reaction conditions: benzyl alcohol (1 mmol), solvent (5 mL), 6 h.

The results indicate that in the absence of the catalyst or with only CQDs-Tpy present, negligible amounts of aldehyde were produced (Table 1, Entries 1 and 2). However, increasing the catalyst amount significantly enhanced the product yields (Table 1, Entries 3–5). The influence of temperature on the reaction was also examined, revealing improved yields of benzaldehyde as the temperature increased from room temperature to 90 °C (Entries 5–7). Different solvents, including an initial H_2_O:EtOH (1:1, v/v) mixture, H_2_O/CH_3_CN solvent mixtures, toluene, and water, were tested, but no significant improvements in yield were observed (Table 1, Entries 8–10). Furthermore, the presence of 1 mmol K_2_CO_3_ increased the yield, but further increasing the amount did not enhance the reaction (Table 1, Entry 12). Additionally, it was observed that the addition of hydrogen peroxide (H_2_O_2_) improved the yields of benzaldehyde in the presence of Pd-based catalyst systems. The yield of benzyl alcohol oxidation increased when H_2_O_2_ was used as the oxidant (Table 1, Entries 13–15). Moreover, increasing the H_2_O_2_/benzyl alcohol ratio from 1:1 to 3:1 resulted in an increase in yield from 65 to 82% at room temperature.

To demonstrate the versatility of this approach, a range of different alcohols were subjected to the optimized reaction conditions to synthesize the corresponding aldehydes, and the outcomes are presented in Table 2. It is worth noting that the oxidation of various benzylic alcohols yielded the corresponding benzaldehyde compounds in high yields (58–89%), without further oxidation to benzoic acid. Electron-donating substituted benzyl alcohols exhibited higher yields and shorter reaction times compared to those with electron-withdrawing substituents (Table 2, Entries 2 and 3). Previous studies have indicated that the rate-determining step in noble metal-catalyzed alcohol oxidation is β-hydride elimination, involving the cleavage of a C-H bond. Given the suggested involvement of carbocation intermediates, this reaction is sensitive to substituent effects. Electron-donating groups provide substantial stabilization to carbocation species, enabling the oxidation of benzyl alcohols bearing such groups at slightly faster rates than those with electron-withdrawing substituents^47^. Notably, both alcoholic groups of 1,4-benzenedimethanol were oxidized successfully using this oxidation protocol (Table 2, Entry 6). The efficacy of the PHC system was also explored for secondary alcohols. The reactions involving secondary alcohols yielded relatively lower yields compared to primary alcohols (Table 2, Entries 7–9). However, despite the efficiency of CQDs-Tpy/Pd NPs in the oxidation of primary and secondary benzylic alcohols, aliphatic alcohols could not be oxidized using this catalyst system (Table 2, Entry 10).

**Table 2:** CQDs-Tpy/Pd NPs catalyzed selective oxidation of alcohols^a^.


Entry	Substrate	Product	Time (h)	Conversion (%)	Selectivity (%)
1			6	85	> 99
2			6	89	> 99
3			6	86	> 99
4			6	81	> 99
5			12	59	> 99
6			12	81	> 99
7			24	63	> 99
8			24	61	> 99
9			24	58	> 99
10		–	24	–	–

^a^Reaction conditions: alcohols (1 mmol), 90 °C, EtOH/H_2_O (5 mL).

**Figure 8:**
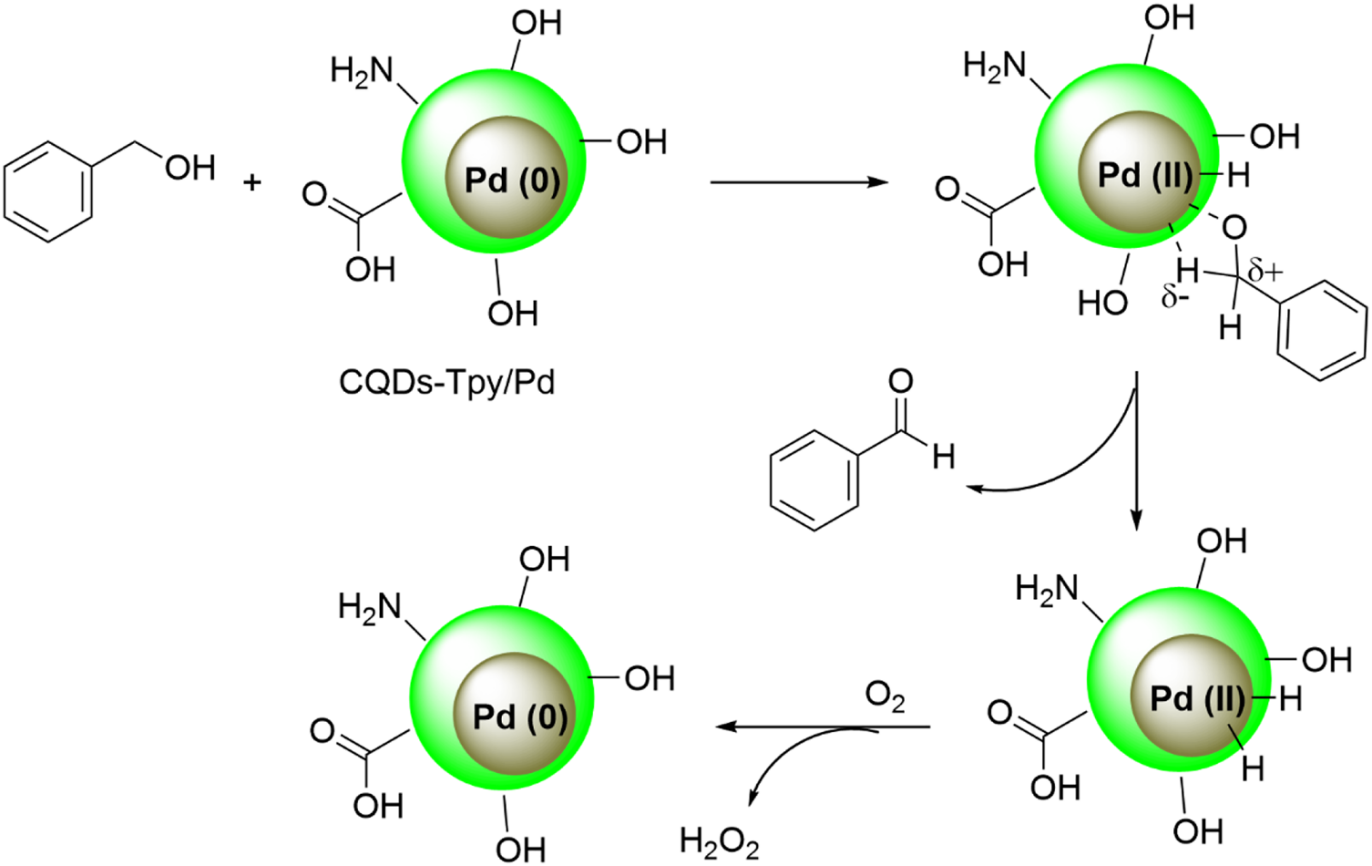
The plausible mechanisms for the aerobic oxidation of alcohol.

The literature suggests plausible mechanisms for the epoxidation of alcohols, as depicted in Fig. 8. The reaction pathway begins with the oxidative addition of the alcohol to the coordinatively unsaturated Pd(0) at the edge of the nanoparticle, resulting in the formation of a Pd(II)-alcoholate intermediate. This intermediate then undergoes β-hydride elimination, leading to the formation of the corresponding carbonyl compound and a Pd-hydride species. The reaction between the Pd(II) hydride and O_2_ regenerates the initial Pd(0) species (Fig. 8), while simultaneously producing H_2_O_2_^47,48^.

**Figure 9:**
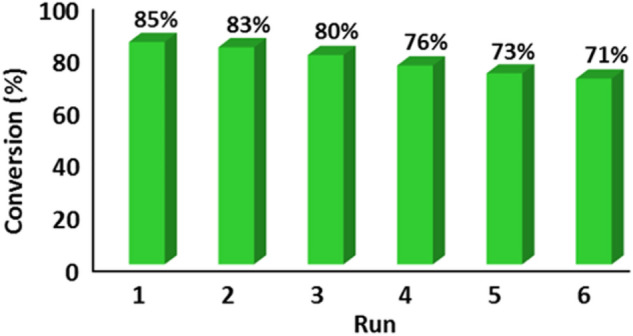
Reusability of CQDs-Tpy/Pd NPs for the oxidation of benzyl alcohol reaction.

Upon completion of the reaction, the reaction mixture was subjected to centrifugation to facilitate the separation of the PHC from the reaction products, enabling its reuse in subsequent runs (95% of the catalyst mass was recovered). Impressively, recycling experiments demonstrated the chemical stability and recyclability of the catalyst, as the recovered catalyst could be successfully employed in at least five consecutive runs (Fig. 9).

A hot-filtration test was carried out in order to confirm the catalyst stability of QCDs-Tpy/Pd NPs. After 150 min of reaction, the catalyst was separated by centrifugation, and its composition was followed for more 360 min. Figure S2 compares the yields of aldehydes obtained from the hot-filtration experiment and the normal catalytic experiment. No significant conversion was observed in this experiment, implying the absence of substantial Pd leaching from QCDs-Tpy/Pd NPs catalyst under reaction conditions used.

To assess the stability of the catalyst structure, the recovered catalyst after six catalytic cycles underwent characterization using various techniques, including ICP, XRD, TEM, and EDX mapping. ICP analysis of the recovered catalyst after five catalytic cycles revealed that 13% of Pd had leached into the reaction medium (the catalyst after the 6th run, containing 0.0218 and 0.0189 mmol of Pd, respectively). Table S1 shows the comparison of catalytic performance of QCDs-Tpy/Pd NPs and Pd content in the recovered catalysts. The XRD pattern of the recovered CQDs-Tpy/Pd NPs demonstrated the preservation of the lattice integrity of the CQDs-Tpy/Pd NPs (Figure S3). Moreover, EDX mapping of the recovered CQDs-Tpy/Pd NPs catalyst provided further confirmation of the structural integrity of the recycled catalyst (Figure S4). TEM analysis was conducted on the recovered CQDs-Tpy/Pd NPs catalyst to gain a better understanding of its morphology. The TEM images of the recovered catalyst, as shown in Figure S5, indicated no observable changes in the morphology of the CQDs-Tpy/Pd NPs catalyst. A comparison between the characterization of the fresh and recovered catalyst clearly demonstrated no significant alterations in its structure, thus highlighting the inherent stability of the synthesized PHC. A comparison of the catalytic performance of the CQDs-Tpy/Pd NPs with various reported Pd-based heterogeneous catalysts is presented in Table 3. The results demonstrate that the PHC exhibits favorable catalytic performance and remarkable selectivity for the oxidation of benzyl alcohol under an air atmosphere when compared to the data available in the literature.

**Table 3:** Catalytic performance of different Pd-based nanocatalysts for selective oxidation of benzyl alcohol.

Catalyst	Conditions	Conv. (%)	Benzaldehyde Select. (%)	Refs.
Pd/NaX zeolite	Toluene, O_2_, 100 °C	66	97	^49^
Pd/SBA-15	*p*-Xylene, O_2_, 120 °C	99	90	^41^
Pd-pol	H_2_O, K_2_CO_3_, air, 100 °C	98	> 99	^47^
Pd/Al_2_O_3_	Solvent free, O_2_, 120 °C	80	94	^50^
Pd/HMSNs ∼Pyra/Pd	EtOH, H_2_O_2_, 60 °C	70	99	^51^
Pd/CNTs	Xylene, air, 90 °C	89	98	^45^
Pd/MagSBA	Solvent free, O_2_, 85 °C	83	80	^52^
AuPd-PVA/TiO_2_	O_2_, 120 °C	72	69	^53^
Pd@Cu(II)-MOF	Xylene, air, 130 °C	95	> 99	^54^
Pd@U-E15	H_2_O, K_2_CO_3_, O_2_, 90 °C	90	90	^55^
CQDs-TPy/Pd NPs	EtOH/H_2_O, K_2_CO_3_ air, 90 °C	85	> 99	This work

## Conclusion

In summary, this study demonstrates the utilization of terpyridine ligand-functionalized carbon quantum dots (CQDs-Tpy) as an effective platform for immobilizing Pd NPs, resulting in the development of a highly efficient PHC. The catalyst demonstrates exceptional performance in selectively oxidizing aromatic alcohols under aerobic conditions, offering the advantages of easy recovery and efficient regeneration. The presence of CQDs-Tpy plays a crucial role in stabilizing the in situ-formed Pd NPs species, which are responsible for the selective oxidation of alcohol derivatives. Notably, the CQDs-Tpy/Pd NPs catalyst achieves remarkable conversion rates (up to 89%) and exhibits excellent selectivity towards aldehydes (> 99%). Furthermore, the catalyst demonstrates favorable catalytic activity and stability, as evidenced by successful recycling and reuse for six cycles without significant loss in performance. Overall, this study presents an improved, environmentally friendly, efficient, and cost-effective oxidative protocol for the selective oxidation of aromatic primary and secondary alcohols to their corresponding aldehydes, with outstanding selectivity. Given the promising catalytic performance of the PHC, we believe it holds great promise for future applications. Ongoing research in our group is focused on further exploring PHC systems.

### Supplementary Information

Supplementary Information.

## Data Availability

All the associated with this work are presented here (and its Supplementary Information file) and further will be made available on reasonable request. Correspondence and requests for materials should be addressed to A.R.

## References

[1] Tojo G, Fernández MI (2006). Oxidation of Alcohols to Aldehydes and Ketones: A Guide to Current Common Practice.

[2] Mallat T, Baiker A (2004). Oxidation of alcohols with molecular oxygen on solid catalysts. Chem. Rev..

[3] Mohammadi M, Rezaei A, Khazaei A, Xuwei S, Huajun Z (2019). Targeted development of sustainable green catalysts for oxidation of alcohols via tungstate-decorated multifunctional amphiphilic carbon quantum dots. ACS Appl. Mater. Interfaces..

[4] da Melo IE (2019). Au−Pd selectivity-switchable alcohol-oxidation catalyst: Controlling the duality of the mechanism using a multivariate approach. ChemCatChem.

[5] Pereira LN (2020). Accessing basic sites on modified CoFe_2_O_4_ nanoparticles: Addressing the selective oxidation of benzyl alcohol and unraveling the Au: Pd ratio effects by XPS. J. Braz. Chem. Soc..

[6] Kopylovich MN (2015). Catalytic oxidation of alcohols: Recent advances. Adv. Organomet. Chem..

[7] Targhan H, Evans P, Bahrami K (2021). A review of the role of hydrogen peroxide in organic transformations. J. Ind. Eng. Chem..

[8] Allen SE, Walvoord RR, Padilla-Salinas R, Kozlowski MC (2013). Aerobic copper-catalyzed organic reactions. Chem. Rev..

[9] Sigman MS, Jensen DR (2006). Ligand-modulated palladium-catalyzed aerobic alcohol oxidations. Acc. Chem. Res..

[10] Torbina VV (2018). Ag-based catalysts in heterogeneous selective oxidation of alcohols: A review. Catalysts.

[11] Amiri R (2022). Carbon quantum dots decorated Ag/CuFe_2_O_4_ for persulfate-assisted visible light photocatalytic degradation of tetracycline: A comparative study. J. Water Process. Eng..

[12] Chen L (2019). Heterogeneous photocatalysis for selective oxidation of alcohols and hydrocarbons. Appl. Catal. B: Environ..

[13] Parmeggiani C, Cardona F (2012). Transition metal based catalysts in the aerobic oxidation of alcohols. Green Chem..

[14] Davis SE, Ide MS, Davis RJ (2013). Selective oxidation of alcohols and aldehydes over supported metal nanoparticles. Green Chem..

[15] Hagen J (2015). Industrial Catalysis: A Practical Approach.

[16] Philippot, K. & Serp, P. Concepts in nanocatalysis. In *Nanomaterials in catalysis*, 1–54 (2013).

[17] Sheldon RA, Arends IW, Dijksman A (2000). New developments in catalytic alcohol oxidations for fine chemicals synthesis. Catal. Today.

[18] Karimi B, Zamani A (2008). Recent advances in the homogeneous palladium-catalyzed aerobic oxidation of alcohols. Journal of the Iranian Chemical Society.

[19] Targhan H, Hassanpour A, Bahrami K (2019). Highly efficient polymer-stabilized palladium heterogeneous catalyst: Synthesis, characterization and application for Suzuki-Miyaura and Mizoroki-Heck coupling reactions. Appl. Organomet. Chem..

[20] Targhan H, Hassanpour A, Sohrabnezhad S, Bahrami K (2020). Palladium nanoparticles immobilized with polymer containing nitrogen-based ligand: A highly efficient catalyst for Suzuki-Miyaura and Mizoroki-Heck coupling reactions. Catal. Lett..

[21] Yang F (2015). Pd/PdO nanoparticles supported on carbon nanotubes: A highly effective catalyst for promoting Suzuki reaction in water. Catal. Today.

[22] Bahrami K, Targhan H (2019). A new strategy to design a graphene oxide supported palladium complex as a new heterogeneous nanocatalyst and application in carbon–carbon and carbon-heteroatom cross-coupling reactions. Appl. Organomet. Chem..

[23] Elazab HA, Siamaki AR, Moussa S, Gupton BF, El-Shall MS (2015). Highly efficient and magnetically recyclable graphene-supported Pd/Fe_3_O_4_ nanoparticle catalysts for Suzuki and Heck cross-coupling reactions. Appl. Catal. A: Gen..

[24] Shen L, Wu W, Liang R, Lin R, Wu L (2013). Highly dispersed palladium nanoparticles anchored on UiO-66 (NH 2) metal-organic framework as a reusable and dual functional visible-light-driven photocatalyst. Nanoscale.

[25] Zhang Y, Li Y-X, Liu L, Han Z-B (2019). Palladium nanoparticles supported on UiO-66-NH2 as heterogeneous catalyst for epoxidation of styrene. Inorg. Chem. Commun..

[26] Biffis A, Zecca M, Basato M (2001). Metallic palladium in the heck reaction: Active catalyst or convenient precursor?. Eur. J. Inorg. Chem..

[27] Jana S, Dutta B, Bera R, Koner S (2008). Immobilization of palladium in mesoporous silica matrix: Preparation, characterization, and its catalytic efficacy in carbon−carbon coupling reactions. Inorg. Chem..

[28] Ramirez E (2004). Influence of organic ligands on the stabilization of palladium nanoparticles. J. Organomet. Chem..

[29] Sobhani S, Zeraatkar Z, Zarifi F (2015). Pd complex of an NNN pincer ligand supported on γ-Fe_2_O_3_@SiO_2_ magnetic nanoparticles: A new catalyst for Heck, Suzuki and Sonogashira coupling reactions. New J. Chem..

[30] Kwak Y, Matyjaszewski K (2009). ARGET ATRP of methyl methacrylate in the presence of nitrogen-based ligands as reducing agents. Polym. Int..

[31] Elahi SM, Raizada M, Sahu PK, Konar S (2021). Terpyridine-based 3D metal–organic-frameworks: A structure-property correlation. Chem. Eur. J..

[32] Adibi-Motlagh B (2022). Immobilization of modular peptides on graphene cocktail for differentiation of human mesenchymal stem cells to hepatic-like cells. Front. Chem..

[33] Rezaei A, Zheng H, Majidian S, Samadi S, Ramazani A (2023). Chiral Pseudohomogeneous Catalyst Based on Amphiphilic Carbon Quantum Dots for the Enantioselective Kharasch–Sosnovsky Reaction. ACS Appl Mater Interfaces..

[34] Rezaei A, Mohammadi Y, Ramazani A, Zheng H (2022). Ultrasound-assisted pseudohomogeneous tungstate catalyst for selective oxidation of alcohols to aldehydes. Sci. Rep..

[35] Rezaei A (2021). Pseudohomogeneous metallic catalyst based on tungstate-decorated amphiphilic carbon quantum dots for selective oxidative scission of alkenes to aldehyde. Sci. Rep..

[36] Hadian-Dehkordi L (2020). Amphiphilic carbon quantum dots as a bridge to a pseudohomogeneous catalyst for selective oxidative cracking of alkenes to aldehydes: A nonmetallic oxidation system. ACS Appl. Mater. Interfaces.

[37] Mohammadi M, Khazaei A, Rezaei A, Huajun Z, Xuwei S (2019). Ionic-liquid-modified carbon quantum dots as a support for the immobilization of Tungstate Ions (WO42–): Heterogeneous nanocatalysts for the oxidation of alcohols in water. ACS Sustain. Chem. Eng..

[38] Rezaei A, Hashemi E (2021). A pseudohomogeneous nanocarrier based on carbon quantum dots decorated with arginine as an efficient gene delivery vehicle. Sci. Rep..

[39] Hadizadeh N (2022). An overview on the reproductive toxicity of graphene derivatives: Highlighting the importance. Nanotechnol. Rev..

[40] Piermatti O (2021). Green synthesis of Pd nanoparticles for sustainable and environmentally benign processes. Catalysts.

[41] Bayan R, Karak N (2017). Photo-assisted synthesis of a Pd–Ag@ CQD nanohybrid and its catalytic efficiency in promoting the suzuki–miyaura cross-coupling reaction under ligand-free and ambient conditions. ACS Omega.

[42] Wang Z (2017). One-step and green synthesis of nitrogen-doped carbon quantum dots for multifunctional electronics. RSC Adv..

[43] Javan H, Asghari E, Ashassi-Sorkhabi H (2019). Fabrication and electrochemical kinetics studies of reduced carbon quantum dots-supported palladium nanoparticles as bifunctional catalysts in methanol oxidation and hydrogen evolution reactions. Synth. Met..

[44] Maity N (2020). Fly ash supported Pd–Ag bimetallic nanoparticles exhibiting a synergistic catalytic effect for the reduction of nitrophenol. Dalton Trans..

[45] Shinde VM, Skupien E, Makkee M (2015). Synthesis of highly dispersed Pd nanoparticles supported on multi-walled carbon nanotubes and their excellent catalytic performance for oxidation of benzyl alcohol. Catal. Sci. Technol..

[46] Guo Z (2014). Recent advances in heterogeneous selective oxidation catalysis for sustainable chemistry. Chem. Soc. Rev..

[47] Dell’Anna MM, Mali M, Mastrorilli P, Cotugno P, Monopoli A (2014). Oxidation of benzyl alcohols to aldehydes and ketones under air in water using a polymer supported palladium catalyst. J. Mol. Catal. A Chem..

[48] Wang Z (2022). Dual Pd2+ and Pd0 sites on CeO_2_ for benzyl alcohol selective oxidation. J. Catal..

[49] Li F, Zhang Q, Wang Y (2008). Size dependence in solvent-free aerobic oxidation of alcohols catalyzed by zeolite-supported palladium nanoparticles. Appl. Catal. A Gen..

[50] Wang X, Wu G, Guan N, Li L (2012). Supported Pd catalysts for solvent-free benzyl alcohol selective oxidation: Effects of calcination pretreatments and reconstruction of Pd sites. Appl. Catal. B Environ..

[51] Ghorbani S, Parnian R, Soleimani E (2021). Pd nanoparticles supported on pyrazolone-functionalized hollow mesoporous silica as an excellent heterogeneous nanocatalyst for the selective oxidation of benzyl alcohol. J. Organomet. Chem..

[52] Li Y (2016). Heterogeneous Pd catalyst for mild solvent-free oxidation of benzyl alcohol. J. Mol. Catal. A Chem..

[53] Wang J (2015). Au–Pd nanoparticles dispersed on composite titania/graphene oxide-supports as a highly active oxidation catalyst. ACS Catal..

[54] Chen G-J (2016). Pd@ Cu (II)-MOF-catalyzed aerobic oxidation of benzylic alcohols in air with high conversion and selectivity. Inorg. Chem..

[55] Karimi B, Khorasani M, Vali H, Vargas C, Luque R (2015). Palladium nanoparticles supported in the nanospaces of imidazolium-based bifunctional PMOs: The role of plugs in selectivity changeover in aerobic oxidation of alcohols. Acs Catal..

