# Supplementary material for: Palladium-based pseudohomogeneous catalyst for highly selective aerobic oxidation of benzylic alcohols to aldehydes
Source: Sci Rep. 2024 Jan 4;14:536. doi: 10.1038/s41598-023-49526-y (PMC10766977; doi:10.1038/s41598-023-49526-y)

#### Supplementary Information

#### Palladium-based Pseudohomogeneous Catalyst for Highly Selective Aerobic Oxidation of Benzylic Alcohols to Aldehydes

Homa Targhan,^€^ Aram Rezaei,^€^* Alireza Aliabadi,^£^* Ali Ramazani,^¥^* Zhefei Zhao,^Þ^ and Huajun Zheng ^Þ^*

€ Nano Drug Delivery Research Center, Health Technology Institute, Kermanshah University of Medical Sciences, Kermanshah, Iran

£ Pharmaceutical Sciences Research Center, Health Institute, School of Pharmacy, Kermanshah University of Medical Sciences, Kermanshah, Iran

¥ Department of Chemistry, University of Zanjan, Zanjan 45371-38791, Iran

Þ Department of Applied Chemistry, Zhejiang University of Technology, Hangzhou 310032, China

Corresponding authors: Aram Rezaei, Email: [aram.rezaei@gmail.com](mailto:aram.rezaei@gmail.com); Alireza Aliabadi, Email: [aliabadi.alireza@gmail.com](mailto:aliabadi.alireza@gmail.com); Ali Ramazani, [aliramazani@gmail.com](mailto:aliramazani@gmail.com); Huajun Zheng, zhenghj@zjut.edu.cn.


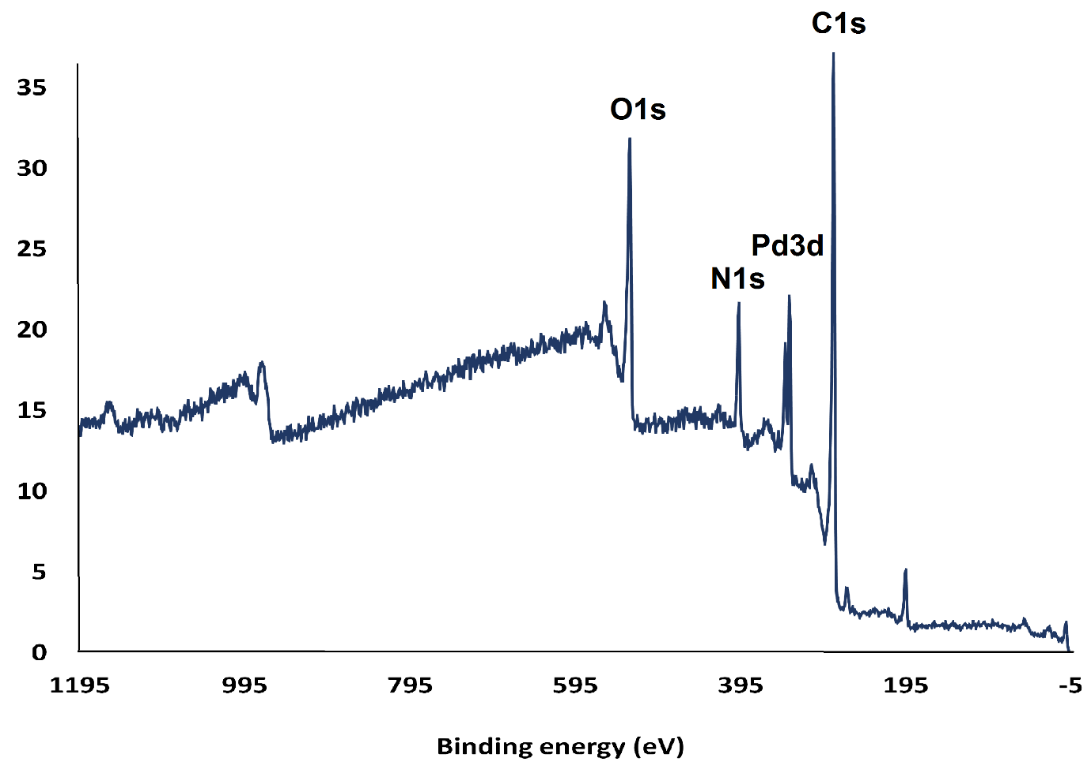


**Figure S1.**  XPS spectral of CQDs-Tpy /Pd NPs

**Figure S2.** Hot filtration test for the selective oxidation of benzyl alcohol with the CQDs-Tpy/Pd NPs catalyst.

**Table S1.** Comparing catalytic performance of QCDs-Tpy/Pd NPs and Pd content in the recovered catalysts

| Entry | Pd content (mmol) in the 0.1 g of QCDs-Tpy/Pd NPs | Conversion (%) |
| --- | --- | --- |
| 1 | 0.0218 | 85 |
| 2 | 0.0211 | 83 |
| 3 | 0.0207 | 80 |
| 4 | 0.0200 | 76 |
| 5 | 0.0195 | 73 |
| 6 | 0.0189 | 71 |


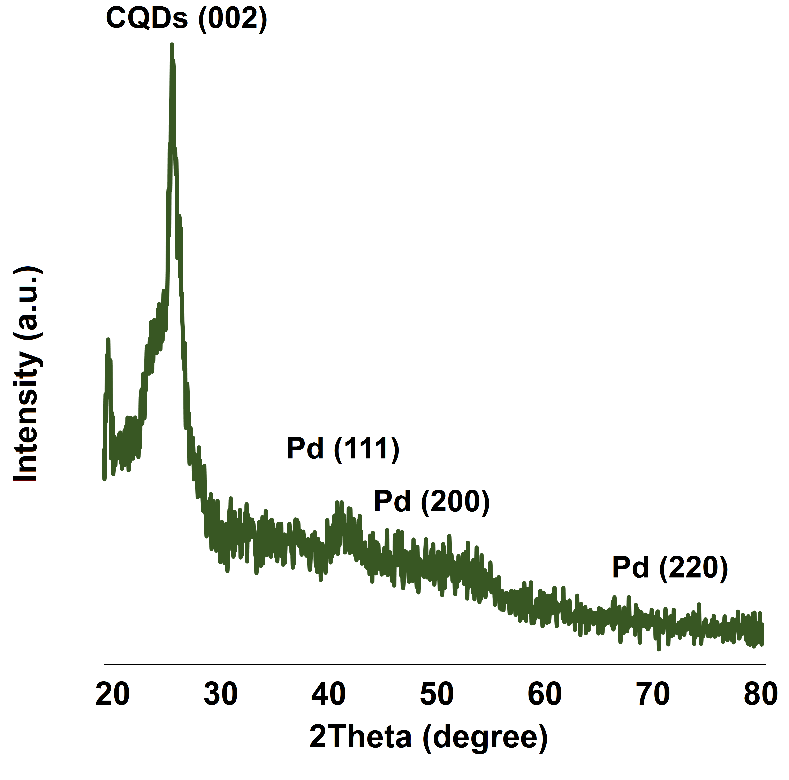


**Figure S3.** XRD pattern of the recovered CQDs-Tpy/Pd NPs


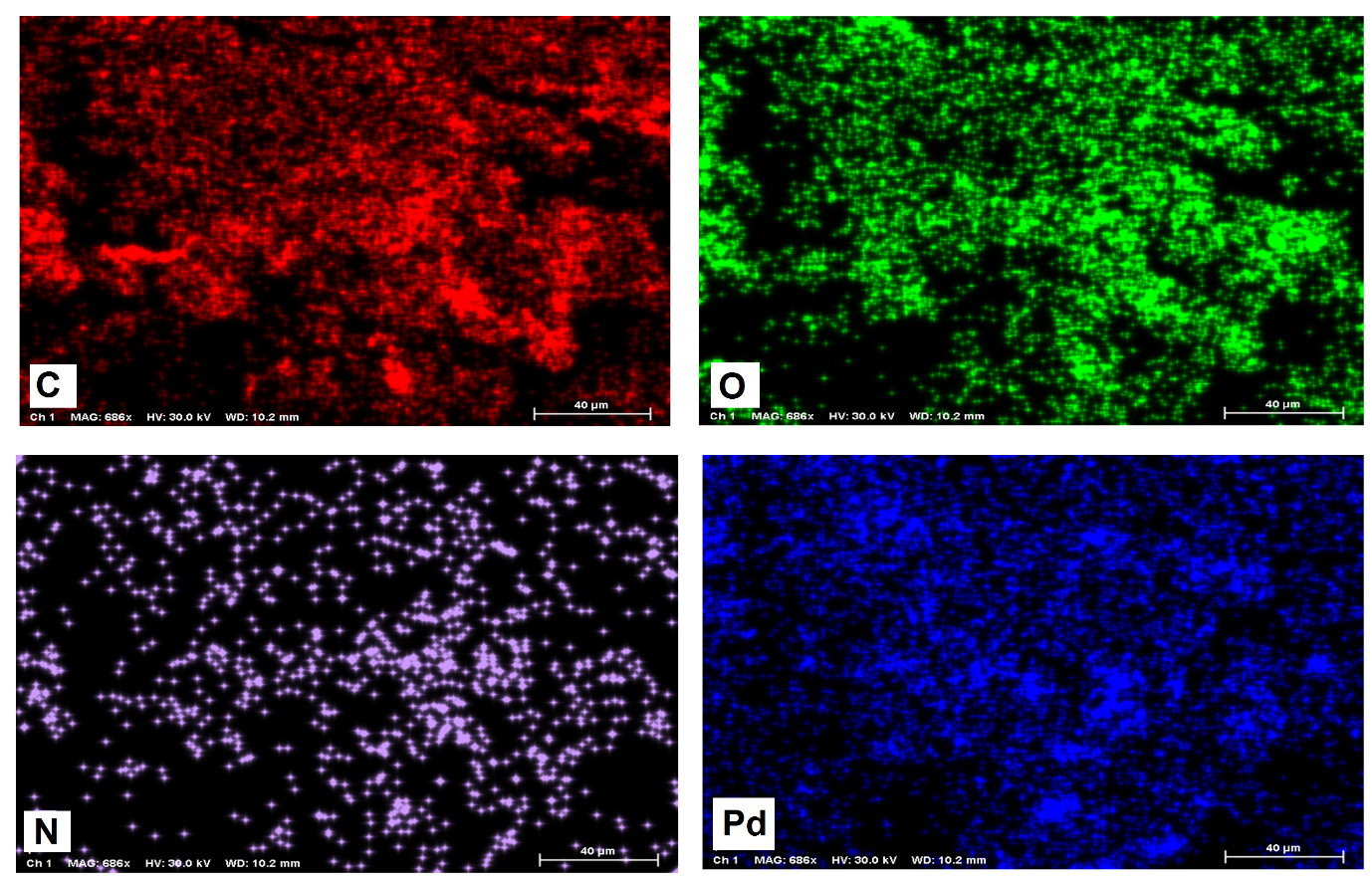


**Figure S4.** EDX mapping of the recovered CQDs-Tpy/Pd NPs


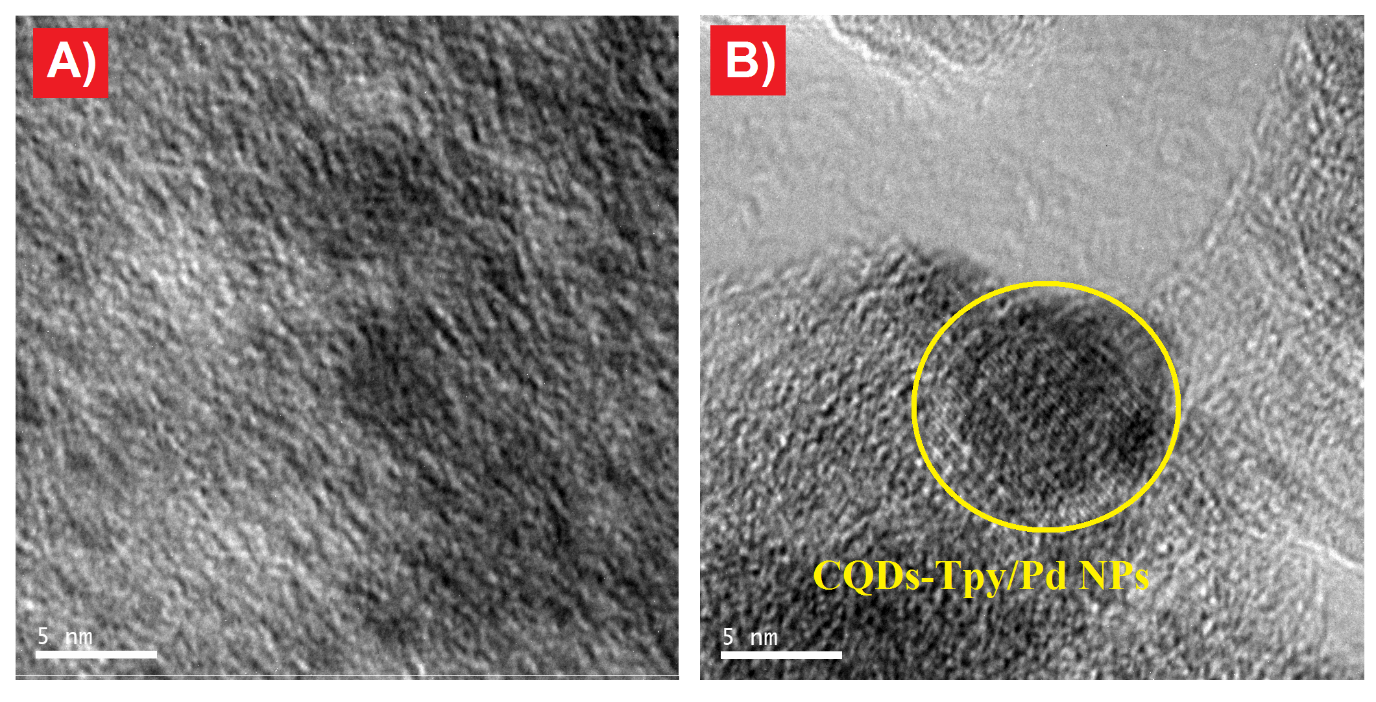


**Figure S5.** TEM images of the recovered CQDs-Tpy/Pd NPs

**^1^HNMR data of the selected products**

**Benzaldehyde (Table 2, entry 1):** ^1^HNMR (250 MHz, DMSO): δ =7.53-7.56 (2H, d, *J* = 7.5 Hz), 7.62-7.64 (1H, t), 7.85-7.88 (2H, d, *J* = 7.5 Hz), 9.98 (1H, s).


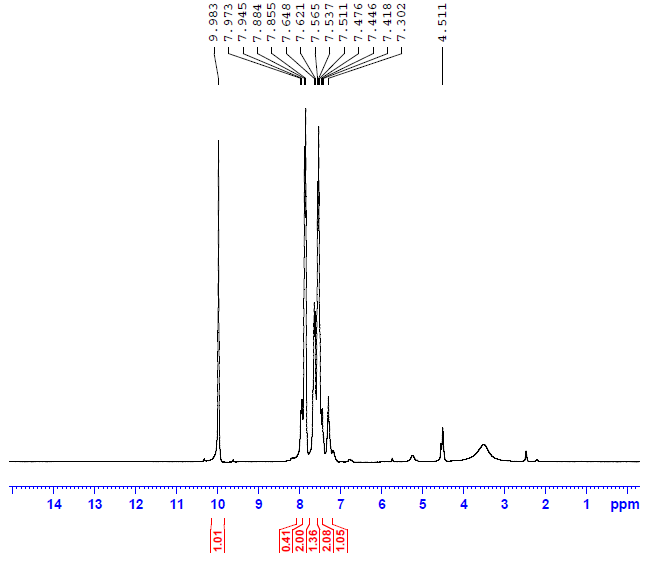

**Terephthalaldehyde** **(Table 2, entry 6):** ^1^HNMR (250 MHz, DMSO): δ =8.08 (4H, s), 10.10 (2H, s).


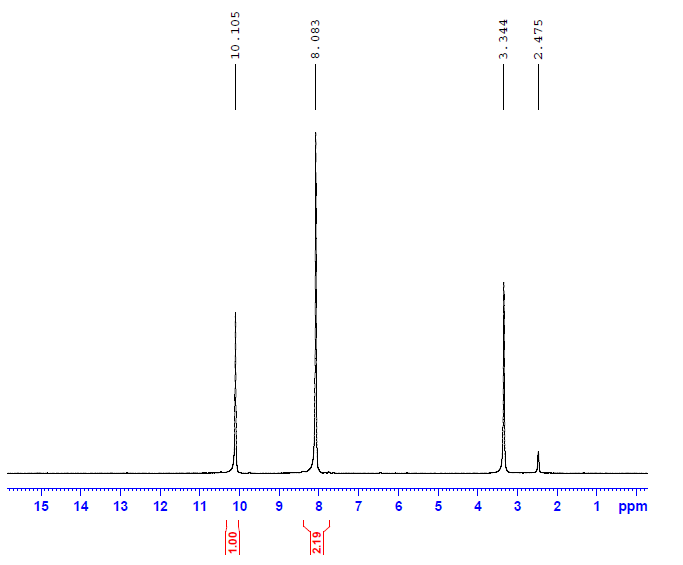

Supplement: Supplementary file 1 — Supplementary Information. [file 41598_2023_49526_MOESM1_ESM.docx]
